# Acidosis as a promising early indicator of mortality among point-of-care parameters and vital signs in non-traumatic critically ill patients

**DOI:** 10.1186/s13049-025-01409-z

**Published:** 2025-05-14

**Authors:** Asen S. Georgiev, Tim Filla, Janina Dziegielewski, Katharina Bandmann, Peter Kienbaum, Jörg Distler, Lennert Böhm, Michael Bernhard, Mark Michael

**Affiliations:** 1 Emergency Department, University Hospital, Medical Faculty, Heinrich-Heine University, Duesseldorf, Germany; 2 Department of Rheumatology, University Hospital, Medical Faculty, Heinrich-Heine University, Duesseldorf, Germany; 3 Hiller Research Center, University Hospital, Medical Faculty, Heinrich-Heine University, Duesseldorf, Germany; 4 Department of Anesthesiology, University Hospital, Medical Faculty, Heinrich-Heine University, Duesseldorf, Germany

**Keywords:** Acidemia, pH value, Blood gas analysis, Emergency care, Outcome

## Abstract

**Background:**

The management of critically ill patients, arriving at the emergency department (ED), requires structured care in critical care facilities, particularly in the resuscitation room. This study examines the significance of initial vital signs and blood gas analysis (BGA)-derived values as clinically useful early indicators of mortality risk in critically ill patients, both during in the resuscitation room care and within the following 30 days, with a focus on evaluating the individual predictive performance of accessible clinical parameters.

**Methods:**

We pooled data from two consecutive retrospective observational studies in a German university ED to analyze an unselected patient population of non-traumatic critically ill patients. Vital signs, such as heart rate, systolic blood pressure, and BGA values (including pH, bicarbonate, carbon dioxide, glucose, lactate, electrolyte levels) on admission to the ED, were used to estimate the impact on both resuscitation room and 30-day mortality.

**Results:**

In 1,536 critically ill patients, pH, lactate and bicarbonate were found to be potential predictors of resuscitation room mortality. In contrast, vital signs showed limited reliability in predicting outcomes. Of all tested variables, pH demonstrated the highest area under the curve (AUC) value among the analyzed markers for resuscitation room mortality (AUC 0.81 [95% CI 0.75–0.87]). However, the AUC of pH for 30-day mortality decreased to 0.64 ([0.6 – 0.68], indicating a complex interplay of factors influencing long-term outcome. A subgroup analysis based on pH showed a substantial increase in resuscitation room and 30-day mortality for patients with a pH below 7.2 as well as a second increase below 7.0.

**Conclusion:**

Our study highlights important parameters for the assessment of critically ill patients at ED admission that are helpful for formulating immediate medical decisions. Acidosis on the initial BGA appears to be a relevant prognostic marker for mortality in critically ill, non-traumatic patients and may aid in early risk assessment, regardless of the underlying condition. Early detection of acidosis could facilitate rapid decision-making and timely identification of patients requiring intensive care.

## Introduction

Up to 15% of patients arriving at the emergency department (ED) fall within the highest two triage categories (red and orange, based on the 5 category Manchester triage system), requiring immediate allocation of medical resources [1]. Furthermore, several studies showed that 1.5–2.0% of all ED patients suffer from non-traumatic critically ill conditions and were treated in the resuscitation room [2, 3]. Although resuscitation room care might commonly be associated with patients who suffer from major trauma, in practice, non-traumatic critically ill patients are up to four times more prevalent [4]. Given the diverse and complex nature of non-traumatic critical conditions—including respiratory failure, circulatory shock, and altered consciousness—early prognostication is essential for optimizing patient management and resource allocation.

Though trauma patients also require assessments for the severity of their condition, the primary cause of trauma can often be rapidly identified through primary and secondary surveys often based on the standardized Advanced Trauma Life Support (ATLS) or ETC (European Trauma Course) methods [5, 6]. The extent of injuries is then further evaluated based on clinical necessity, including targeted imaging such as a full-body computed tomography (CT) scan when indicated. For many non-traumatic critically ill patients, the completion of diagnostics is usually required to identify the cause of the current critical conditions. These can span the entire spectrum of emergency medicine (e.g., respiratory failure, shock, cardiac arrest, reduced vigilance) [7], so that the initial clinical picture rarely is conclusive, as individual factors are often unclear (e.g. pre-existing conditions). Hence, in such emergency situations, decisions often need to be made in a universal setting without any prior medical history. In such high-stakes situations, clinicians must rely on immediate clinical assessments and objective parameters to estimate the severity of the patient’s condition.

Given these challenges, assessing a critically ill patient’s survival prospects upon arrival at the resuscitation room is crucial. Understanding the survival prospects of critically ill patients upon their arrival at the resuscitation room in an ED holds pivotal importance in guiding immediate medical interventions, resource allocation and the triage process. Rapid and informed decision-making is paramount for each critically ill non-traumatic patient.

Previous studies have emphasized the significance of markers like initial lactate and glucose levels in the resuscitation room, which serve as potential indicators for mortality risk across various admission reasons [8–10]. However, despite their valuable insight, there is a need to comprehensively compare these markers with alternatives, like pH-value, to evaluate individual parameters for their potential as predictors of mortality in the resuscitation room.. This exploratory analysis aims toevaluate the individual prognostic performance of traditional and “alternative” markers in this critical setting. Recognizing the most effective markers not only aids in risk stratification but also ensures that critically ill patients are identified at the earliest possible stage, allowing planning of adequate further care.

Furthermore, early detection of critically ill patients is vital for improving outcomes, as it facilitates timely admission to the intensive care unit (ICU) [11], where patients can receive the necessary intensive monitoring and intervention. Recognizing the severity of illness, clinicians can prevent delays in critical care, leading to improved survival rates in these high-risk populations. The aim of this investigation was to examine the significance of initial vital signs and blood gas analysis (BGA)-derived values as potential indicators of mortality risk in critically ill patients, both during in the resuscitation room care and within the following 30 days.

## Methods

We analyzed the data of two consecutive retrospective, single-center observational studies [Observation of critically ill patients in the resuscitation room of the Emergency Department in Duesseldorf (OBSERvE-DUS 1 and 2)- studies] conducted from March 1, 2018 to February 29, 2020, set in the ED at the university hospital of Duesseldorf, Germany. The study protocol was approved by the Ethics Committee of the Medical Faculty of the Heinrich Heine University of Duesseldorf, Germany (Study Nr. 2023–2535).

### Setting

The university hospital's service area encompasses the city of Duesseldorf, Germany, with approximately 650,000 residents. During the study period, about 45,000 patients were treated in the ED annually, of which approximately 60% presented non-traumatic acute diseases or emergencies. Critical care facilities include four specialized resuscitation rooms for the treatment of severely injured or critically ill patients, facilitating immediate intensive medical procedures such as airway management, mechanical ventilation, cardiovascular therapy, and invasive circulation monitoring. These interventions are concurrently recorded in the patient data management system (PDMS) by the treating ED physicians and nurses. Critically ill non-traumatic patients are treated in these resuscitation rooms by a team comprising two nurses, one resident physician, and one attending physician specialized in emergency and intensive care, with other specialists brought in as needed and in accordance with national recommendations [12].

### Study definitions and data collection

The objective of this study was to evaluate different parameters to determine which may serve as practical and universally applicable indicators of mortality risk in critically ill, non-traumatic patients. These parameters needed to be obtainable in every patient, regardless of the availability of clinical history, presenting symptoms, known pre-existing conditions, or ongoing medical interventions. Unlike scoring systems or complex calculations, which may introduce delays or require additional clinical input, the chosen parameters should be straightforward to interpret and applicable across all patient groups without discrimination. Most importantly, it should provide a direct estimation of the severity of a patient’s condition by correlating with the probability of mortality, both during resuscitation room care and within 30 days of admission, thereby offering a rapid and actionable tool for early risk assessment in emergency settings.

All adult patients aged 18 and above, who were critically ill and non-traumatic, and were admitted to the ED resuscitation room, were enrolled. Epidemiological and medical care information had been previously anonymized and collected in the OBSERvE-DUS 1 and 2 studies (data published partially elsewhere [3], data from OBSERvE-DUS 2 study not published) using the PDMS (COPRA®, COPRA System GmbH, Berlin, Germany) and hospital information system (MEDICO®, Cerner Deutschland GmbH, Itstein, Germany) through database querying, and were subsequently transferred to a spreadsheet program (Microsoft® Office 365, version 16.37, Microsoft Corporation, Redmond, USA). In compliance with data protection regulations under the German Data Protection Regulation (DSGVO) and adherence to Good Clinical Practice (GCP) guidelines, only anonymized data was analyzed so that individual patients could not be identified.

Patients were included in this study via a step-by-step identification process involving treatment in one of the four accessible resuscitation rooms, meeting at least one criterion from our resuscitation room admission list [systolic blood pressure (SBP) < 90 mmHg or oxygen saturation (SpO2) < 90% or Glasgow coma scale (GCS) < 15 points)] [13], and manual review of medical records. Detailed information on data collection as well as inclusion and exclusion criteria can be found in the OBSERvE-DUS 1 and 2 studies (3). This resulted in an inclusion of a total of 1,536 individuals. Of note, 275 patients had missing data about 30-day survival due to transfer to another hospital or prior discharge with no information about their future status. For this reason, the patient cohort for the 30-day mortality consisted of 1261 patients. Notably, the patients who did not survive the resuscitation room care were included in the cohort of patient who did not survive 30 days after admission, since they were considered “admitted” at their arrival at the resuscitation room.

The evaluation chart of the ED resuscitation room encompassed anthropometric characteristics such as age, gender, weight, and height. Triage categorization and vital signs upon ED admission in the resuscitation room [e.g., SBP (in mmHg), heart rate (HR) (x/min), respiratory rate (RR) (x/min), level of consciousness measured using GCS (points), and SpO2 (%)] were documented. As part of the routine resuscitation room protocol, the first blood gas analysis (BGA)—including the admission blood lactate and electrolyte level—was documented (blood gas analyzer: ABL800 FLEX XQ, Radiometer Medical ApS, Bronshoj, Denmark). Of note, the blood gas analyzer performed the calculations using a standard temperature of 37 degrees Celsius (98.6 degrees Fahrenheit). In total 335 of the BGAs were arterial, since studies have shown no distinction between arterial and venous BGA for the parameters of interest [14, 15], venous and arterial BGAs were combined. Furthermore, no follow up BGAs or other parameters were taken into account. Primary ABCDE (airway, breathing, circulation, disability, environment) problem was derived from the Emergency Medical Service (EMS) protocol and are shown in Tables 1 and 2. The final diagnoses were grouped in the following seven main categories according to the systematic of the previously published OBSERvE studies (3) abdominal emergencies, cardiovascular emergencies, metabolic disorders, neurological emergencies, pulmonary emergencies, sepsis/infection and other emergencies and the subsequent subgroups as shown in Table 3. The diagnoses were obtained from the discharge papers. For the patients who did not survive, the most likely cause of death was taken. Of note, patients with a diagnosis of STEMI already during the pre-hospital care were taken directly to our cardiac catheterization laboratory. Diagnosis of STEMI in our resuscitation room was included into the analysis as it was initially treated there before going to the catheter lab. Due to initial misdiagnosis, a very small number of trauma patients (0.2% of the whole patient population) were included as they were initially treated as critically ill non-traumatic patients.

**Table 1:** Patient´s characteristics, vital signs, and blood gas analysis of resuscitation room survivors and non-survivors

**Number of patients [n, (%)]**	**Overall **1,536 (100.0)	**Non-survivor **67 (4.4)	**Survivor **1,469 (95.6)	***p*-value**
**Patient’s characteristics (MV ± SD)**				
Age (years)	69.7 ± 16.2	75.6 ± 13.1	69.5 ± 16.3	**0.003**
Sex, female [n, (%)]	717 (46.7)	32 (47.8)	685 (46.6)	0.847
Weight (kg)	78.5 ± 22.3	77.9 ± 18.1	78.5 ± 22.4	0.829
BMI (points)	25.0 ± 7.2	23.2 ± 4.6	25.0 ± 7.2	**0.043**
**ABCDE problems [n, (%)]**				
A (airway)	34 (2.2)	0 (0.0)	34 (2.3)	0.210
B (breathing)	398 (25.9)	16 (23.9)	382 (26.0)	0.701
C (circulation)	459 (29.9)	44 (65.7)	415 (28.3)	**< 0.001**
D (disability)	606 (39.5)	4 (6.0)	602 (41.0)	**< 0.001**
E (environment)	39 (2.5)	3 (4.5)	36 (2.5)	0.313
**Vital signs admission [MV ± SD]**				
Systolic blood pressure (mmHg)	129.9 ± 44.5	104.1 ± 41.6	130.7 ± 44.3	**< 0.001**
Heart rate (bpm)	95.7 ± 31.5	96.8 ± 34.3	95.7 ± 31.4	0.780
Shock index (bpm/mmHg)	0.9 ± 0.5	1.0 ± 0.6	0.8 ± 0.5	**0.002**
Oxygen saturation (%)	93.9 ± 7.2	89.9 ± 12.0	94.0 ± 6.9	**< 0.001**
Respiratory rate (min^−1^)	21.4 ± 10.1	32.3 ± 27.9	21.0 ± 8.5	**< 0.001**
Glasgow coma score (points)	11.0 ± 4.8	6.8 ± 5.1	11.1 ± 4.7	**< 0.001**
Temperature tympanal (°C)	36.2 ± 1.5	35.4 ± 2.5	36.3 ± 1.4	**< 0.001**
**BGA parameters [MV ± SD]**				
pH	7.31 ± 0.15	7.09 ± 0.25	7.32 ± 0.14	**< 0.001**
pCO_2_ (mmHg)	48.7 ± 19.8	66.1 ± 30.9	47.9 ± 18.8	**< 0.001**
HCO^−^_3_ (mmol/l)	21.8 ± 5.6	16.4 ± 10.1	22.1 ± 5.2	**< 0.001**
Hemoglobin (g/dl)	12.9 ± 2.7	11.8 ± 2.2	12.9 ± 2.8	**0.002**
Lactate (mmol/l)	3.5 ± 3.6	9.2 ± 6.4	3.3 ± 3.2	**< 0.001**
Glucose (mg/dl)	182.8 ± 114.7	256.0 ± 169.8	179.7 ± 110.8	** < 0.001**
Na^+^ (mmol/l)	138.6 ± 6.3	139.3 ± 8.6	138.6 ± 6.2	0.376
K^+^ (mmol/l)	4.4 ± 1.0	5.1 ± 2.2	4.3 ± 0.9	** < 0.001**
Ca^2+^ (mmol/l)	1.2 ± 0.1	1.2 ± 0.1	1.2 ± 0.1	1.000
**Procedures [n, (%)]**:				
catecholamine	465 (30.3)	41 (61.2)	424 (28.9)	** < 0.001**
cardiopulmonary resuscitation	96 (6.2)	41 (61.2)	55 (3.7)	** < 0.001**

*BGA* Blood gas analysis, *MV* Mean value, *SD* Standard deviation, *BMI* Body mass index, *mmHg* millimeter(s) of mercury, *bpm* beats per minute, *pCO*_*2*_ partial pressure of carbon dioxide, *HCO*^*−*^_*3*_ bicarbonate, *Na*^*+*^ sodium, *K*^*+*^ potassium, *Ca2*^*+*^ calcium

**Table 2:** Patient´s characteristics, vital signs, and blood gas analysis of critically ill patients on 30-day survival

**Number of patients [n, (%)]**	**Overall **1,261 (100.0)	**Non-survivor **297 (23.6)	**Survivor **964 (76,4)	***p*-value**
**Patient’s characteristics (MV ± SD)**				
Age (years)	69.6 ± 16.4	74.9 ± 13.9	67.9 ± 16.8	**< 0.001**
Sex, female [n, (%)]	585 (46.4%)	139 (46.8%)	446 (46.3%)	0.880
Weight (kg)	78.5 ± 21.8	75.6 ± 20.7	79 ± 22	0.0184
BMI (points)	25.1 ± 7	23.7 ± 5.7	25.6 ± 7.2	**< 0.001**
**ABCDE problems [n, (%)]**				
A (airway)	34 (2.7%)	4 (1.3%)	30 (3.1%)	0.093
B (breathing)	294 (23.3%)	62 (20.9%)	232 (24.1%)	0.255
C (circulation)	409 (32.4%)	128 (43.1%)	281 (29.1%)	**< 0.001**
D (disability)	492 (39.0%)	96 (32.3%)	396 (41.1%)	0.007
E (environment)	32 (2.5%)	7 (2.4%)	25 (2.6%)	0.710
**Vital signs admission (MV ± SD)**				
Systolic blood pressure (mmHg)	129.8 ± 44.5	116.3 ± 47.2	133.5 ± 43	**< 0.001**
Heart rate (bpm)	96 ± 32.8	98.8 ± 30.3	95.3 ± 33.4	0.107
Shock index (bpm/mmHg)	0.85 ± 0.48	1.02 ± 0.57	0.81 ± 0.45	**< 0.001**
Oxygen saturation (%)	93.9 ± 7.3	92.6 ± 8.6	94.2 ± 6.9	**0.001**
Respiratory rate (min^−1^)	21.4 ± 10.4	23.8 ± 15.4	20.8 ± 8.4	**< 0.001**
Glasgow coma score (points)	11.1 ± 4.8	8.46 ± 5.0	11.8 ± 4.4	**< 0.001**
Temperature tympanal (°C)	36.2 ± 1.5	35.9 ± 1.9	36.3 ± 1.4	**< 0.001**
**BGA parameters (**MV ± SD)				
pH	7.31 ± 0.15	7.24 ± 0.21	7.34 ± 0.12	**< 0.001**
pCO_2_ (mmHg)	48 ± 19.3	51.4 ± 23.4	47 ± 17.7	**< 0.001**
HCO^−^_3_(mmol/l)	21.6 ± 5.6	19.2 ± 7.3	22.3 ± 4.8	**< 0.001**
Hemoglobin (g/dl)	12.8 ± 2.8	12.4 ± 2.7	12.9 ± 2.8	0.007
Lactate (mmol/l)	3.7 ± 3.7	6.1 ± 5.3	2.9 ± 2.7	**< 0.001**
Glucose (mg/dl)	182.1 ± 113.1	204.2 ± 126.3	175.7 ± 108.0	**< 0.001**
Na^+^ (mmol/l)	138.6 ± 6.3	138.8 ± 8.1	138.5 ± 5.7	0.476
K^+^ (mmol/l)	4.4 ± 1	4.6 ± 1.4	4.3 ± 0.8	**< 0.001**
Ca^2+^ (mmol/l)	1.2 ± 0.1	1.2 ± 0.1	1.2 ± 0.1	1.000
**Procedures [n, (%)]**:				
catecholamine	358 (28.4%)	152 (51.2%)	206 (21.4%)	**< 0.001**
cardiopulmonary resuscitation	84 (6.7%)	68 (22.9%)	16 (1.7%)	**< 0.001**

*BGA* Blood gas analysis, *MV* Mean value, *SD* Standard deviation, *BMI* Body mass index, *mmHg* millimeter(s) of mercury, *bpm* beats per minute, *pCO*_*2*_ partial pressure of carbon dioxide, *HCO*^*−*^_*3*_ bicarbonate, *Na*^*+*^ sodium, *K*^*+*^ potassium, *Ca2*^*+*^ calcium

**Table 3:** Summary of final diagnoses for critically ill non-traumatic patients by resuscitation room and 30-day outcomes

Final Diagnosis:	Total		Res-Surv		Non-Res-Surv		30 d-Surv		Non-30 d-Surv	
Absolute/Percentage:	**n**	**%**	**n**	**%**	**n**	**%**	**n**	**%**	**n**	**%**
**Abdominal Emergencies**	**73**	**4.8**	**70**	**4.6**	**3**	**0.2**	**41**	**3.3**	**23**	**1.8**
Bowel Ischemia	7	0.5	7	0.5	0	0.0	2	0.2	5	0.4
Gastrointestinal Bleeding	41	2.7	39	2.5	2	0.1	26	2.1	11	0.9
Ileus (Intestinal Obstruction)	1	0.1	1	0.1	0	0.0	0	0.0	1	0.1
Other Emergencies	24	1.6	23	1.5	1	0.1	13	1.0	6	0.5
**Cardiovascular Emergencies**	**398**	**25.9**	**355**	**23.1**	**43**	**2.8**	**249**	**19.7**	**108**	**8.6**
Ruptured Aortic Aneurysm	5	0.3	5	0.3	0	0.0	1	0.1	3	0.2
Cardiac Arrest	104	6.8	71	4.6	33	2.1	33	2.6	59	4.7
Hypertensive Emergency	18	1.2	18	1.2	0	0.0	16	1.3	0	0.0
Hypotension	6	0.4	6	0.4	0	0.0	5	0.4	1	0.1
Cardiac Decompensation	90	5.9	89	5.8	1	0.1	65	5.2	12	1.0
Cardiogenic Shock	36	2.3	32	2.1	4	0.3	13	1.0	20	1.6
Pulmonary Embolism	15	1.0	13	0.8	2	0.1	9	0.7	5	0.4
Pulmonary Edema	19	1.2	18	1.2	1	0.1	15	1.2	1	0.1
Myocardial Infarction	12	0.8	10	0.7	2	0.1	5	0.4	4	0.3
Arrhythmia	84	5.5	84	5.5	0	0.0	81	6.4	1	0.1
Other Emergencies	3	0.2	3	0.2	0	0.0	3	0.2	0	0.0
Thrombosis	1	0.1	1	0.1	0	0.0	1	0.1	0	0.0
Type A Aortic Dissection	5	0.3	5	0.3	0	0.0	2	0.2	2	0.2
**Metabolic Disorders**	**61**	**4.0**	**60**	**3.9**	**1**	**0.1**	**43**	**3.4**	**8**	**0.6**
Acute Kidney Failure	6	0.4	6	0.4	0	0.0	3	0.2	2	0.2
Electrolyte Imbalance	12	0.8	11	0.7	1	0.1	6	0.5	4	0.3
Hepatic Encephalopathy	8	0.5	8	0.5	0	0.0	5	0.4	1	0.1
Hyperglycemia	18	1.2	18	1.2	0	0.0	14	1.1	0	0.0
Hypoglycemia	7	0.5	7	0.5	0	0.0	7	0.6	0	0.0
Cardiorenal Syndrome	10	0.7	10	0.7	0	0.0	8	0.6	1	0.1
**Neurological Emergencies**	**495**	**32.2**	**490**	**31.9**	**5**	**0.3**	**316**	**25.1**	**86**	**6.8**
Idiopathic Parkinson's Syndrome	1	0.1	1	0.1	0	0.0	1	0.1	0	0.0
Intracerebral Hemorrhage	79	5.1	76	4.9	3	0.2	26	2.1	43	3.4
Ischemia	306	19.9	305	19.9	1	0.1	216	17.1	31	2.5
Seizure	96	6.3	95	6.2	1	0.1	70	5.6	4	0.3
Meningitis	3	0.2	3	0.2	0	0.0	0	0.0	3	0.2
Altered mental status	6	0.4	6	0.4	0	0.0	3	0.2	1	0.1
Cerebral Hypoxia	1	0.1	1	0.1	0	0.0	0	0.0	1	0.1
Cerebral Mass	3	0.2	3	0.2	0	0.0	0	0.0	3	0.2
**Pulmonary Emergencies**	**117**	**7.6**	**113**	**7.4**	**4**	**0.3**	**78**	**6.2**	**9**	**0.7**
COPD	112	7.3	108	7.0	4	0.3	74	5.9	8	0.6
Pneumothorax	4	0.3	4	0.3	0	0.0	4	0.3	0	0.0
Pulmonary Bleeding	1	0.1	1	0.1	0	0.0	0	0.0	1	0.1
**Sepsis/Infection**	**266**	**17.3**	**256**	**16.7**	**10**	**0.7**	**149**	**11.8**	**52**	**4.1**
Foreign Body Infection	2	0.1	2	0.1	0	0.0	2	0.2	0	0.0
Pneumonia	186	12.1	180	11.7	6	0.4	97	7.7	40	3.2
Unclear	15	1.0	12	0.8	3	0.2	7	0.6	5	0.4
Urosepsis	56	3.6	55	3.6	1	0.1	38	3.0	5	0.4
Soft Tissue Infection	7	0.5	7	0.5	0	0.0	5	0.4	2	0.2
**Other Emergencies**	**126**	**8.2**	**125**	**8.1**	**1**	**0.1**	**88**	**7.0**	**11**	**0.9**
Anaphylaxis	29	1.9	29	1.9	0	0.0	29	2.3	0	0.0
Acute Bleeding	6	0.4	6	0.4	0	0.0	6	0.5	0	0.0
Dehydration	3	0.2	3	0.2	0	0.0	3	0.2	0	0.0
Hyperthermia	2	0.1	2	0.1	0	0.0	2	0.2	0	0.0
Hypothermia	5	0.3	4	0.3	1	0.1	2	0.2	2	0.2
Intoxication	62	4.0	62	4.0	0	0.0	37	2.9	4	0.3
Pressure Ulcer	5	0.3	5	0.3	0	0.0	2	0.2	1	0.1
Mental Disorder	1	0.1	1	0.1	0	0.0	0	0.0	0	0.0
Other Emergencies	10	0.7	10	0.7	0	0.0	5	0.4	3	0.2
Traumatic injury	3	0.2	3	0.2	0	0.0	2	0.2	1	0.1
**Grand Total**	**1536**	**100.0**	**1469**	**95.6**	**67**	**4.4**	**964**	**76.4**	**297**	**23.6**

*Res-Surv* Survivors of resuscitation room treatment, *Non-Res-Surv* Non-survivors of resuscitation room treatment, *30 d-Surv* survivors 30 days after resuscitation room treatment, *Non-30-Surv *non survivors 30 days after resuscitation room treatment

From this data, we selected several quantifiable parameters and correlated them to patients’ outcome. Parameters were chosen based on the speed and ubiquity of their availability (within 15 min of admission to the resuscitation room; available in > 90% of patients). They included anthropomorphic characteristics (gender, age), vital signs (HR, SBP, SpO2), and BGA parameters [pH-value, lactate, bicarbonate (HCO3), glucose, hemoglobin (Hb), sodium (Na +), potassium (K +), calcium (Ca2 +)]. Tympanic temperature, GCS and respiratory rate were initially included in the cohort description but ultimately excluded as potential prognostic parameters due to their absence in more than 10% of patient data.

Furthermore, even though it is primarily used for assessment of hemorrhagic shock in trauma patients, we added the so-called “shock index (SI)” as a variable and derived it by dividing patients’ HR by their SBP. The use of catecholamines or cardiopulmonary resuscitation (CPR) was not taken into account in the analysis. Diastolic blood pressure and diastolic shock index were omitted from our analysis as we considered SBP and SI to be more representative of a patient’s hemodynamic status in the acute setting.

As mentioned in the study definition above, we did not exclude patients based on additional variables such as presenting symptoms, pre-existing medical conditions, specific diagnoses, pre-hospital interventions, or underlying metabolic disturbances, including different types of acidosis. By maintaining an inclusive approach, we aimed to reflect real-world clinical scenarios in which critically ill patients present with undifferentiated conditions requiring immediate intervention. In line with this approach, we did not differentiate between SARS-CoV-2 positive and negative patients.

### Statistical analysis and subgroup definition

In the cohort description, descriptive statistics were provided as absolute numbers, while proportions were used for binary and nominal covariates. For continuous covariates, mean and standard deviation were used. To assess the predictive power on resuscitation room and 30-day mortality, the area under the curve (AUC) with 95% bootstrap confidence intervals (CI) was independently calculated for each variable. These included nine blood gas analysis-derived values, along with age, gender, SBP, heart rate, shock index, and oxygen saturation as independent variables. The outcomes were either resuscitation room mortality or 30-day mortality (yes/no). This study did not include formal statistical comparison of AUC values (e.g., DeLong test), as the primary aim was to explore individual parameter performance rather than establish definitive ranking. Given the exploratory nature of the study and the high likelihood of multicollinearity among several parameters, we chose not to perform multivariable logistic regression, as it may have produced unstable estimates and limited interpretability. Furthermore, gray-zone analysis (90% sensitivity, specificity) was calculated for each. For each clinical marker, the optimal cutoff point for survival decision-making was determined by maximizing the Youden-index where sensitivity and specificity were equally weighted. In order to gain a more detailed understanding of how pH is related to resuscitation room or 30-day mortality, the mortality rate with exact 95% CI was calculated for specific pH categories. All statistical analyses were performed using R version 4.2 [16] and illustrations were created using DataGraph (Version 4.6.1, Visual Data Tool Inc., Chapel Hill, NC, USA).

We divided the whole study population into the following subgroups: resuscitation room survivors (Res-Surv) vs. resuscitation room non-survivors (Non-Res-Surv), as well as 30-day survivors (30 d-Surv) vs. 30-day non-survivors (Non-30 d-Surv). Furthermore, we divided the patients into eight different groups based on their pH-value (Table 4).

**Table 4:** Probability of dying in the resuscitation room and within 30 days based on pH value

pH value	Prevalence in resuscitation room (%)	Probability dying resuscitation room (%, 95% CI)	Probability dying within 30 days (%, 95% CI)
< 6.80	1.2	47.1 (25.4–69.7)	94.1 (75.6–99.0)
6.80—6.99	3.3	21.3 (11.5–34.5)	48.6 (33.2–64.0)
7.00—7.09	3.8	7.4 (2.6–16.7)	37.8 (24.7–52.0)
7.10–7.19	8.3	12.7 (7.6–19.6)	39.8 (30.3–50.0)
7.20–7.29	16.2	3.9 (2.0–7.0)	28.2 (22.0–35.0)
7.30–7.39	40.4	1.4 (0.7–2.6)	15.8 (12.8–19.0)
7.40–7.49	23.3	1.2 (0.4–2.8)	18.7 (14.3–24.0)
> 7.50	3.5	0.0 (0.0–4.9)	12.5 (4.9–25.0)

## Results

The patient cohort had a mean age of 69.7 ± 16.2 years, with 46.7% being female. Anthropometric characteristics, vital signs, and results of BGA upon ED admission were detailed in Tables 1 and 2. The mortality rate in the resuscitation room was 4.4%. The vital signs of Non-Res-Surv showed statistically significant differences to the Res-Surv group, specifically, for the parameters SBP, SpO2, and RR (Table 1). In comparison to Res-Surv group patients, Non-Res-Surv group patients had lower pH and HCO3 levels, and higher pCO2, lactate, and glucose levels (Table 1).

Of the patients with available survival outcome data on day 30, 23.6% did not survive to 30 days. The vital signs of the Non-30 d-Surv also notably differed from the 30 d-Surv (Table 2). Among Non-30 d-Surv group patients, RR, HR, pCO_2_, lactate, and glucose levels were higher, whereas SBP, SpO_2_, tympanal temperature, pH, and bicarbonate levels were lower compared to the 30 d-Surv group (Table 2).

**Fig. 1:**
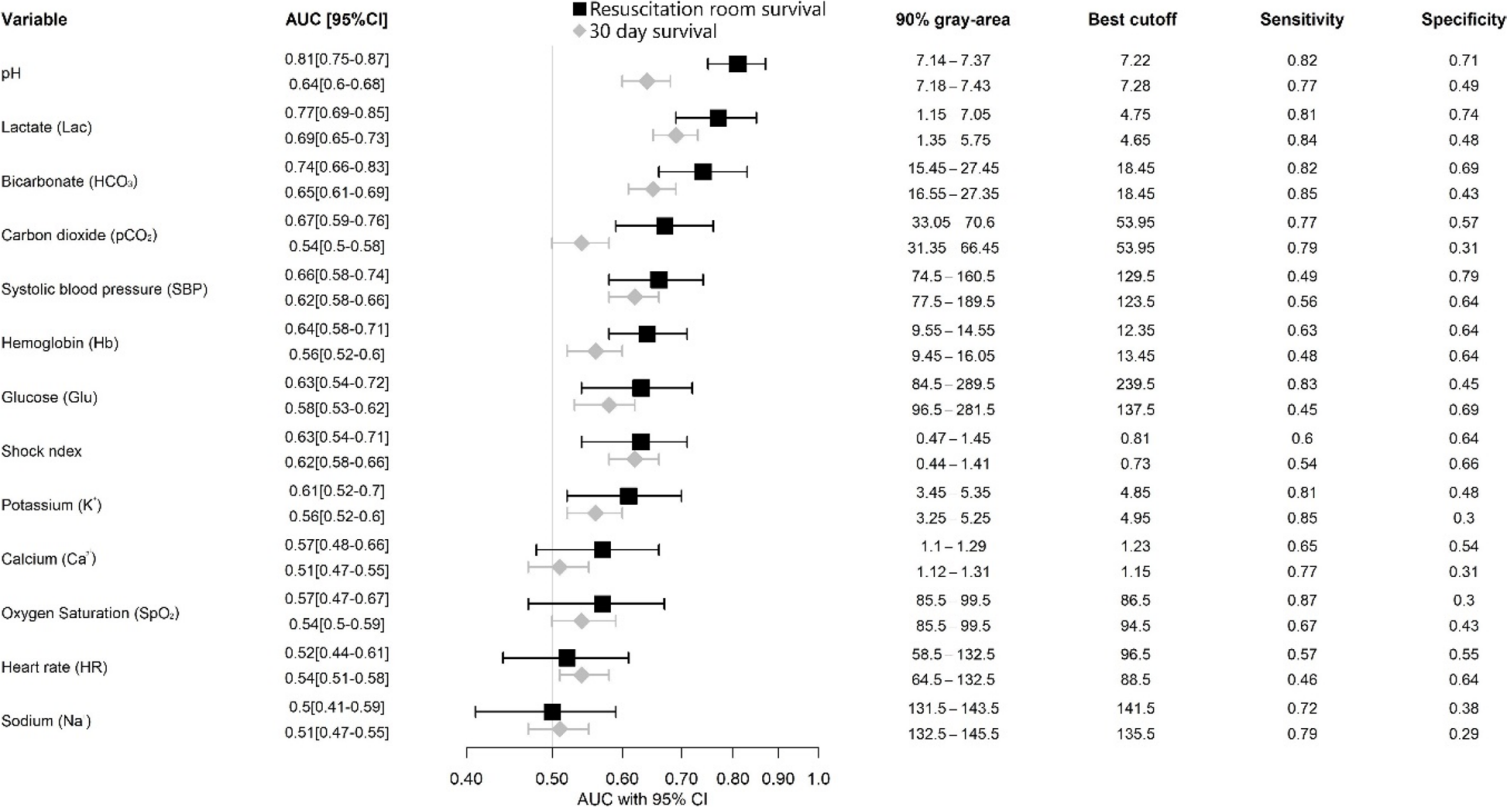
Forest plot for survival of resuscitation room and 30-day survival, including the AUC value with a 95% confidence interval as well as a 90% gray and the optimal cutoff point (determined by using Youden index and with their sensitivity and specificity values respectively)

The AUC values for the different parameters for resuscitation room treatment mortality are described in Fig. 1. Of all markers, the variables with the highest AUC value were pH-value (AUC: 0.81, 95% CI: 0.75–0.87), lactate (AUC: 0.77, 95% CI: 0.69–0.85), and bicarbonate (AUC: 0.74, 95% CI: 0.66–0.83). In comparison, the AUC value of these three markers for 30-day mortality prediction was lower, with no values over 0.7 (Fig. 1). Nevertheless, the AUC value of these markers was still the highest overall, with lactate having the highest AUC (0.69, 95% CI: 0.65–0.73), followed by bicarbonate (0.65, 95% CI: 0.61–0.69) and pH-value (0.64, 95% CI: 0.6–0.68).

The 90% gray-area in Fig. 1 represents the range of values where the predictive ability of each parameter is most uncertain. This interval highlights the overlap between survival and non-survival groups, indicating reduced discriminatory power within this range. For example, the pH-value's 90% gray-area spans 7.14 to 7.37, meaning that while values outside this range are more predictive, those within it provide less certainty in distinguishing outcomes. Similarly, lactate (1.15–7.05) and bicarbonate (15.45–27.45) exhibit broad gray areas, reflecting variability in their predictive performance. Additionally, the best cutoff values for these markers indicate the optimal thresholds for distinguishing between survival and non-survival with equal importance given to both specificity and sensitivity. The best cutoff for pH is 7.22, with a sensitivity of 0.82 and specificity of 0.71, indicating its potential as a clinically relevant predictor of mortality. Lactate’s optimal cutoff of 4.75 (sensitivity 0.81, specificity 0.74) and bicarbonate’s cutoff of 18.45 (sensitivity 0.82, specificity 0.69) further reinforce their relevance in resuscitation room mortality prediction, serving as valuable clinical decision points.

For the pH-subgroup analysis, two groups (7.30–7.39 and 7.40–7.49) were considered to represent normal pH-value ranges and, as expected, did not differ considerably concerning the mortality rate (resuscitation room 1.4% for 7.30–7.39 vs. 1.2% for 7.40–7.49, and 30-day 15.8% for 7.30–7.39 vs. 18.7% for 7.40–7.49) (Table 4). As pH decreased, both resuscitation room mortality as well as 30-day mortality increased (Table 4). One exception was seen in the group 7.00–7.09, which had a similar outcome to the group 7.10–7.19 in resuscitation room mortality with 7.4% (95% CI: 2.6–16.7%) and 12.7% (95% CI: 7.6–19.6%) respectively, as well as 30-day mortality with 37.8% (95% CI: 24.7–52.0%) and 39.8% (95% CI: 30.3–50.0%), respectively. The group with a pH lower than 6.80 had the highest mortality, with resuscitation room mortality at 47.1% and 30-day mortality at 94.1%. Alkalosis (pH > 7.50) did not show a worse outcome than the normal range, but the comparison is limited due to the relatively low proportion (3.5% of the cohort) of patients in the alkalosis group.

## Discussion

This retrospective analysis of data from two observational studies conducted at the university hospital of Duesseldorf, Germany, with a patient cohort of more than 1,500 patients aimed to evaluate the predictive power of various clinical and laboratory parameters in assessing mortality risk among critically ill non-traumatic patients in the resuscitation room. The evaluation of this data offers important first insights into critically ill non-traumatic patient prognosis, independent of external variables such as presenting symptoms, suspected diagnosis, patient characteristics, or preexisting conditions.

Existing research has already examined the performance of several predictive tools, usually in the form of scores, such as quick sequential organ failure assessment (qSOFA) [17], rapid emergency medicine score (REMS) [18, 19], rapid acute physiology score (RAPS) [19], and modified early warning score (MEWS) [20], which are able to predict the mortality of different populations of critically ill non-traumatic patients, with varying degrees of success. However, these scores are primarily limited to vital signs and level of consciousness, because these objective patient characteristics are usually immediately discernible. In recent years, the increased availability in hospital resuscitation rooms of point-of-care BGA, including blood electrolytes, offers a potential addition beyond the vital signs for patients’ risk assessment. Studies on the potential prognostic value of initial blood glucose [9] and lactate [10, 21] levels of critically ill non-traumatic patients have already shown promising results, solidifying these markers as ‘red flags’ in the context of resuscitation room medical care.

Our findings support previous research on lactate [10] with corroborating evidence that high lactate levels correlate with increased mortality, both in the resuscitation room and within the 30-day period following ED admission. Bicarbonate, which is directly linked to acid–base balance, also shows promising results with a solid AUC value of 0.74 (95% CI: 0.66–0.83) for resuscitation room mortality.

Acidosis, measured by a patient’s blood pH value, has already shown promising results as a predictive marker for mortality in intensive care patients [22]. In the ED setting, data points to increased mortality in patients with acidosis [23] and research already exists on patients with extreme acidosis (pH < 6.9) on admission to the ED, showing that they can survive extreme acidosis [24, 25], though it remains unclear if the mortality continuously increases with decrease of pH. In our retrospective analysis, the notably high AUC value for pH of 0.81 (95% CI: 0.75–0.87) highlights its potential prognostic utility for mortality during initial treatment in resuscitation room scenarios. The ROC analysis demonstrated that both pH and lactate showed promise as a prognostic indicator for short-term mortality, reflecting their physiological significance as indicators of cellular dysfunction and metabolic derangement. However, this study did not aim to establish the superiority of one parameter over the other but rather to highlight their individual prognostic value. Given the critical nature of early decision-making in the resuscitation room, recognizing both markers as independent predictors allows for greater clinical flexibility in assessing patient severity. A formal statistical comparison between pH and lactate could provide further insight into their relative predictive strengths and represents a potential direction for future research. Ongoing research into the dynamic role of pH in resuscitation room treatments, including its potential interactions with concurrent treatments and immediate post-resuscitation complications, presents a promising avenue for further investigation. Enhancing the predictive precision of pH, alongside complementary markers, might greatly aid in triage decisions and optimizing emergency care interventions for improved immediate survival outcomes.

In contrast, while pH values remain a key parameter for predicting resuscitation room mortality, their predictive ability over a period of 30 days is less pronounced, indicating a more complex interplay of factors influencing survival beyond the immediate resuscitation phase. Understanding the evolving nature of pH's prognostic value over a 30-day period, while taking into account potential influences of ongoing treatment effects or post-resuscitation complications, offers a direction for further investigation. By integrating dynamic variables and considering the evolving patient status, the predictive utility of pH could be strengthened for extended prognostication in emergency care settings. However, a more comprehensive analysis involving larger cohorts and extensive clinical data is warranted to further understand the precise role of pH in predicting 30-day mortality following initial resuscitation.

The predictive value of pH as a mortality indicator may be time-dependent, with its prognostic strength diminishing as a patient progresses beyond the initial critical phase. Studies suggest that early changes in pH levels, particularly within the first 24 h of ICU admission, can serve as reliable indicators of patient outcomes. For instance, research has shown that the rate of pH change over time correlates with mortality risk, emphasizing the importance of early metabolic assessment [25]. However, this time-dependent degradation does not affect resuscitation room mortality, as pH is measured at the earliest stage of emergency care when its predictive value is most pronounced. This underscores the role of early pH assessment as a rapid, actionable tool for evaluating the severity of critically ill patients upon ED admission, even if its long-term prognostic accuracy may decline over time.

Certain conditions, such as but not limited to diabetic ketoacidosis (DKA) and lactic acidosis, may have different prognostic outcomes due to their distinct pathophysiological mechanisms and response to treatment. DKA, for instance, is characterized by severe metabolic acidosis with profound alterations in pH, yet with timely intervention, it generally has a lower mortality rate compared to other forms of critical illness. In contrast, lactic acidosis often reflects systemic hypoperfusion and is frequently associated with conditions such as sepsis or cardiogenic shock, which carry a clinically relevant higher mortality risk. However, our study focused on the initial phase of emergency care, where such specific diagnoses may not yet be clearly established. Upon arrival in the resuscitation room, critically ill patients present with undifferentiated metabolic and hemodynamic abnormalities, and early decision-making is guided primarily by initial vital signs and metabolic parameters rather than definitive diagnoses, especially when further information about the patient, like previous medical history, is absent. The immediate priority in this setting is stabilization and rapid intervention, regardless of the underlying etiology.

Dividing the non-traumatic critically ill patients into subgroups proved fruitful, as marked differences in mortality rates were observed between groups. A dip of pH below 7.20 notably correlates with escalated mortality rates (Table 4), emphasizing the urgency of prompt intervention, such as volume resuscitation and repletion of potassium deficits in the appropriate cases (e.g., expected intracellular potassium shift after treatment of diabetic ketoacidosis) [26].

In the ED, the focus on pH proves to be pivotal. Elevated pH values above 7.50 do not correlate with worse survival prognosis compared to normal ranges (exact percentage points shown in Table 4), hinting at a different pattern of response or adaptation. However, the pronounced association between lowered pH and escalated mortality highlights the urgency of promptly addressing acidosis. The clinical significance lies in recognizing the threshold levels (pH-value < 7.20; < 7.00) where mortality sharply increases and necessitates immediate intervention strategies. Still, the clinical application remains contingent upon considering other concurrent factors and their interplay with pH-value.

While a combination of variables, such as pH and lactate, may enhance prognostic accuracy, it would also complicate the initial assessment of a patient’s condition in the resuscitation room. Early evaluation in critically ill patients must be rapid and straightforward to avoid delays in critical interventions. Introducing multiple parameters for mortality estimation could result in additional loss of valuable time and increase the risk of miscalculations, particularly in the high-pressure environment of initial stabilization. In contrast, relying on a single, readily available parameter minimizes the risk of human error and allows for immediate interpretation, ensuring that clinical decisions can be made swiftly and efficiently. Furthermore, the type of acidosis – respiratory or metabolic, lactic or alactic may provide the same benefit. From our perspective the advantage of evaluating the pH level, without any further context can provide physicians with an immediate educated “gut feeling” about the severity of the patient in such a stressful, quickly progressing setting such as resuscitation room care. Still, further research of more complex estimates of mortality of non-traumatic, critically ill patients in the resuscitation room or within 30 days, would undoubtedly provide benefits regarding urgent patient care.

Furthermore, our findings could potentially be valuable for healthcare settings that utilize point-of-care testing for blood analysis, as they offer a practical approach to identifying critically ill patients early. By recognizing key prognostic markers such as pH, healthcare providers across various sectors could promptly initiate or escalate emergency medical care, ensuring timely interventions for high-risk patients.

### Limitations

One limitation of this study is its monocentric nature, having been conducted within a tertiary care hospital setting, as this setting might not represent the broader spectrum of the average population. Tertiary care hospitals often attract and handle more severe or complex cases including resuscitation/post-resuscitation care, potentially skewing the dataset towards more critical conditions. As a result, the extrapolation of these findings to more diverse or less critical patient populations seen in different healthcare settings might be limited. Thus, while the study provides important insights, its monocentric design within a specialized care facility needs consideration when applying its findings to broader healthcare contexts.

Given the broad scope of assessed variables and the complexity of potential interactions between them, this study should be considered exploratory in nature. While the findings provide valuable insights into early prognostic markers, they do not establish causal relationships but rather highlight associations that may warrant further investigation in future studies. Moreover, an interaction effect analysis or exploration of non-linear relationships between the different parameters was not performed. While such an analysis could provide deeper insights into potential interdependencies between variables, our has a relatively low number of patients who did not survive both the resuscitation room treatment and the following 30 days. This restricted the statistical power needed for robust interaction effect analysis. Future studies with larger cohorts and a higher number of events would be better suited to investigate these potential interactions and their implications for patient outcomes.

Furthermore, pre-hospital and in-hospital life-saving emergency interventions such as resuscitation, catecholamine use, and invasive airway management have not been taken into account in this analysis, and their impact on the predictive value of the above-mentioned parameters therefore remains unexplored. Other limitations include small sample sizes of the non-survivor subgroups that originate from the scarcity of individuals who do not survive resuscitation room interventions, which constitute only a minute fraction of the overall patient population. Similarly, there exists a relatively low proportion of patients presenting with exceedingly low pH values, further constraining the available pool for analysis. Therefore, the uneven distribution of patients across pH ranges limits the generalizability of outcomes in these critical scenarios. Finally, the study's scope might benefit from exploring a wider array of variables, such as underlying conditions or treatments or an interplay of multiple variables. By gaining more insight into the interplay of pH with other complementary markers, it could be possible to enhance the predictive accuracy of pH values on patient mortality in the resuscitation room and to develop a more effective prognostic score.

## Conclusion

This study identified several clinically relevant parameters for estimating the chances of survival for critically ill, non-traumatic patients upon arrival in the resuscitation room of an ED. These parameters can therefore inform decision-making regarding emergency interventions, resource allocation, and patient triage. pH value appears to be a particularly promising prognostic marker, and it is possible that measuring the pH value during pre-hospital care by the emergency medical services can assist correct allocation to a resuscitation room. The study results emphasize the benefit of early BGA in critically ill non-traumatic patients in the ED, regardless of the suspected diagnosis, and point to acidosis as a reliable early warning signal. In summary, early detection of physiological imbalances through biomarkers such as acidosis, combined with rapid triage to intensive care, is essential for improving patient outcomes. This proactive approach ensures that critically ill patients receive the life-saving interventions they need at the earliest possible stage, ultimately reducing mortality and improving long-term recovery.

## Data Availability

No datasets were generated or analysed during the current study.

## Abbreviations

30 d-Surv: 30-Day survivors
AUC: Area under the curve
BGA: Blood gas analysis
Bpm: Beats per minute
Ca^2+^: Calcium
CT: Computed tomography
DSGVO: German Data Protection Regulation
ED: Emergency department
EMS: Emergency medical service
GCP: Good Clinical Practice
GCS: Glasgow coma scale
Hb: Hemoglobin
HCO_3_^−^: Bicarbonate
HR: Heart rate
K^+^: Potassium
Na^+^: Sodium
Non-30 d-Surv: 30-Day non-survivors
Non-Res-Surv: Resuscitation room non-survivors
PDMS: Patient data management system
Res-Surv: Resuscitation room survivors
RR: Respiratory rate
SBP: Systolic blood pressure
SpO_2_: Oxygen saturation
